# Readiness of managers and health care workers for e-Health: a cross-sectional study in Khartoum primary health care centers, Sudan

**DOI:** 10.1186/s12913-023-10450-6

**Published:** 2023-12-12

**Authors:** Eilaf Yassen, Ibrahim A. Atia, Gaffar Alemam A. Manhal, Mohammedelhadi Elzein, Mozan Mohamed, Musab Siddig, Raghad Eltayeb, Rofida Asmally, Sara Mohammed, Sujood Elhassan

**Affiliations:** Faculty of Medicine, University of Khartoum, Khartoum, Sudan

**Keywords:** e-Health, Readiness, Primary health care, Sudan

## Abstract

**Background:**

e-Health is defined as “the use, in the health sector, of digital data— transmitted, stored and retrieved electronically—for clinical, educational and administrative purposes, both at the local site and at a distance”. In Primary health care (PHC), the role of e-Health in promoting PHC systems defines its need to achieve the PHC aims. This literary work aims to study the readiness of managers and healthcare workers for e-Health at Khartoum state PHC centers.

**Methods:**

This was a facility-based cross-sectional study that took place between February and August 2022. A sample size of 327 was calculated, and multistage cluster sampling was used. A validated questionnaire was used, and the generated data were analyzed using the Statistical Package for Social Sciences (SPSS). Variables were described as n (%) and mean ± SD. Non-parametric tests and Spearman’s correlation were used to investigate the association of readiness scores with different categorical and numerical variables, respectively. A standard multiple regression model was used to model the associations.

**Results:**

A total of 262 forms were completed. The overall readiness percentages were low for both managers (52.8%) and healthcare workers (55.3%). Factors associated with e-Health readiness included occupation, doctors’ level of expertise, PHC center, and PHC center type.

**Conclusions:**

This study reports low levels of e-Health readiness as reflected by managers and HCWs in Khartoum State PHC. Project planners need to be aware of the obstacles and threats faced by e-Health initiatives if they are not carefully planned, executed, and monitored. Special attention must be given to addressing health inequities and inequalities to ensure that these projects will contribute to fostering accessibility to health services and narrowing the digital divide.

**Supplementary Information:**

The online version contains supplementary material available at 10.1186/s12913-023-10450-6.

## Background

The World Health Organization Regional Office for the Eastern Mediterranean defined e-Health as “the use, in the health sector, of digital data— transmitted, stored and retrieved electronically—for clinical, educational and administrative purposes, both at the local site and a distance” [1]. In a broader scope, the Journal of Medical Internet Research adds: “The term characterizes not only a technical development, but also a state-of-mind, a way of thinking, an attitude, and a commitment for networked, global thinking, to improve health care locally, regionally and worldwide by using information and communication technology (ICT)” [2].

In the context of Primary health care (PHC), e-Health helps in promoting system responsiveness to patients’ needs and expectations [3], as well as improving healthcare workers’ (HCWs) relationships with patients [4]. This is consequential because the need to achieve PHC aims is defined with patient-centeredness as a critical value [5]. Furthermore, the urgent need for e-Health projects at the PHC level is justified by the diverse healthcare services provided and the overwhelming amount of health information [6].

Sudan’s health system adopts a district-based approach to health care delivery that asseverates PHC principles [7]. Decentralization in Sudan has led to a significant deterioration of the PHC system, exacerbated by the inequitable allocation of government health expenditure on secondary and tertiary health facilities [7]. Although the implementation of e-Health has shown favorable results for addressing health system challenges at various levels and settings [8], it had by no means enough attention in Sudan’s strategic plan (2017–2020) [9], nor was it considered in recent policy briefs for PHC reform [7].

Several challenges are known to hinder the adoption of e-Health programs in developing countries, including Sudan. These challenges include poor infrastructure, insufficient funds, and the absence of clear national policies and strategies [10, 11]. Moreover, healthcare workers’ unwillingness to adopt e-health (due to lack of experience, ICT literacy, and security and privacy concerns) poses significant challenges to its implementation [11].

Following the recent COVID-19 pandemic, e-Health activation has become one of the major concerns among health planners, including those in developing countries. This is due to its functionality under the social distancing protocols and its great potential for combating similar health situations [12, 13]. It is agreed that e-Health planning and application must be preceded by careful understanding of the situation through performing an e-Health readiness assessment [14]. To this end, this article aims to study the readiness of managers and healthcare workers for e-health application at PHC centers in Khartoum State, and to understand some of the factors that may influence their readiness.

## Methods

### Study design

This was a facility-based, cross-sectional study in Khartoum State PHC centers. Khartoum State is administratively divided into seven localities: Khartoum, Ombada, Omdurman, Karary, Bahri, Sharq Elnil, and Jabal Awliya [15]. Primary health care is delivered within the state borders through 242 PHC centers. Center distribution is roughly equal between the localities, with a mean of 34.5 centers in each of the seven localities. A task force of 7021 individuals works for primary care at different levels. Of these, 6402 represent the workers at the 242 PHC centers [16].

The study proposal was approved by the Khartoum State Ministry of Health - Research Ethics Committee. The study was carried out following the principles of the Declaration of Helsinki. Each study participant was clearly informed about the objectives of the study, and written informed consent was obtained.

### Sample size and sampling

The following formula was used to calculate the study sample size:

$$n=\frac{N}{1+\left(N-1\right){e}^{2}}\times deff$$

Where:

e = level of precision.

N = population size.

deff = design effect.

Considering a 7% level of precision, a design effect of 1.5, and a known population size of 6402 PHC workers, the sample size was found to be 297. Considering a non-response rate of 10%, the sample size of the study was estimated to be 327 PHC workers.

We used a multi-stage cluster sampling technique to select the 327 PHC workers, considering a known average of nine workers per center. Stage one was the selection of three of the seven Khartoum state localities. Each of the three cities in Khartoum state —Khartoum, Omdurman, and Bahri— was considered a stratum. One locality was chosen from each city using simple random sampling. Stage two was the selection of the PHC centers in each locality, using probability proportional to size. In stage three, we selected one manager and eight workers from each PHC center using simple random sampling. We provide a map created using Google Maps services to represent the selected PHC centers and their distance from the center of Khartoum State in supplementary file 1.

### Study tool

Data were collected using a validated, structured, closed-ended, and self-administered questionnaire. The questionnaire was specifically developed for the purpose of assessment of e-Health readiness in developing countries [14]. The original development study was conducted in Pakistan and the final tool was found to be both valid and reliable [14]. The study questionnaire consists of four categories for the managers’ and healthcare workers’ assessment tools (supplementary file 2). Each category consists of several statements (items) evaluated by the participant on a 5-point Likert scale (from strongly disagree to strongly agree).

Three of the four questionnaire categories formed a part of both the manager and the HCW assessment. First, the core readiness category (21 items) addressed matters related to e-Health planning and participants’ experience with ICT Second, societal readiness (11 items) focused on inter-institutional interactions and determinants of service accessibility. Third, policy readiness (12 items) focused on relevant policies at both institutional and governmental levels for launching e-Health projects [14].

The managers’ assessment tool also included a category for technological readiness (10 items) to address the availability and affordability of needed hardware and software for e-Health programs. On the other hand, learning readiness (6 items) formed the fourth category in the health care workers’ tool. It focused on ICT use in health workers’ training [14]. Two experts reviewed the validity and appropriateness of the questionnaire translation carried out by the authors.

In addition to the readiness assessment statements, study variables included participants’ demographics. These include: gender, age, level of education, occupation, employment status, number of years of practice, and duration of employment at the particular PHC center. These variables were gathered from proposals made by experts and pertinent reports on e-Health and associated factors [17]. To demonstrate how the distance from the state’s center influences the readiness scores, an extra variable was included: the “center’s distance from Khartoum State center”.

To encourage consistency in the study participants’ understanding of the concept, the consent form and questionnaire defined e-Health as “the use of modern information and communication technologies to meet the needs of citizens, patients, healthcare professionals, healthcare workers, as well as policymakers” [17].

### Participant selection and data collection

The study inclusion criteria included all workers registered in Khartoum state PHC centers. Cleaning service providers were not included.

The data collection period extended from February to August 2022. The study authors visited the selected centers after obtaining administrative permission from PHC administrative units in the three designated localities. Self-administered forms were distributed among the selected participants. The authors visited each center multiple times to ensure that all handed forms were appropriately completed.

### Data management and analysis

Data were cleaned using Microsoft Excel 2016. Data were then transported and analyzed using the Statistical Package for Social Sciences (SPSS version 27). Cleaned study data are provided as supplementary file 3. The respondents’ characteristics were presented as numbers (%) and mean ± SD. Cronbach’s alpha test was used to independently assess the reliability of questionnaire items for each category. The frequency of each scale point from strongly disagree to strongly agree was presented as a number (%).

Furthermore, numerical values were assigned to the 5-point scale, ranging from 0 for “strongly disagree” to 4 for “strongly agree. Mean scores were calculated for each item to reflect the concepts being assessed and to allow for comparison between categories, as well as the items in each category. Category mean scores were calculated by the sum of the category items’ mean scores. Total readiness scores were calculated using the sum of category scores for managers and healthcare workers. Mean scores were used to calculate category and total readiness percentages.

Low-scoring items were defined as those with less than 50% agree/strongly agree, more than 30% neutral, more than 20% disagree/strongly disagree, or a mean less than 2. In addition, total and category readiness percentages of less than 60% were considered low.

Data were checked for normality, and nonparametric tests, namely Mann-Whitney U and Kruskal-Wallis tests, were used to compare readiness scores across binomial and polynomial variable groups, respectively. *P*-values of less than 0.05 were considered statistically significant. Moreover, Spearman’s Rho correlation was used to investigate the association between the readiness scores and the numerical variables, including: age, PHC center distance from Khartoum State center, total years of practice, and total years of practice at the respective PHC center.

Furthermore, standard multiple regression analysis was used to measure the variance in e-Health readiness scores among the above-mentioned independent variables.

## Results

### Respondents characteristics

Of the 333 forms distributed, 262 forms were filled out, yielding a response rate of 79%, with 11.1% being PHC center managers. The respondents’ mean age was 42.8 (± 9.4). Most of the study participants were females (84%), and more than 40% of the participants did not hold a Bachelor’s degree. Administration and finance employees constituted the largest portion of the participants (29.6%), followed by doctors (17.9%), lab technicians (16.3%) and nurses (12.8%). Details of the respondents’ characteristics are presented in Table 1.

**Table 1:** Respondents’ characteristics (n = 262)

Gender: n (%)	
Male	40 (15.3%)
Female	221 (84.7%)
**Age: (**mean ± SD)	41.8 ± 9.4
**Educational level: n (%)**	
Secondary School	47 (18%)
Technical School	21 (8%)
Diploma	43 (16.5%)
Bachelor’s degree	98 (37.5%)
Master’s degree	42 (16.1%)
Others	10 (3.8%)
**Occupation: n (%)**	
Doctor:	46 (17.9%)
National service	1 (2.2%)
General practitioner	17 (37.8%)
Registrar	18 (40.0%)
Specialist	8 (17.8%)
Consultant	1 (2.2%)
Pharmacist	22 (8.6%)
Nurse	33 (12.8%)
Lab technician	42 (16.3%)
Administration/Finance	76 (29.6%)
Midwife	14 (5.4%)
Vaccination technician	7 (2.7%)
Nutritionist	6 (2.3%)
Others	11 (4.3%)
**Job title at the respective PHC center: n (%)**	
Manager	29 (11.1%)
Health care provider	233 (88.9%)
**Employment status at the respective PHC center: n (%)**	
Part time	88 (34.2%)
Full time	169 (65.8%)
**Total years of practice (**mean ± SD)	15.8 ± 9.5
**Total years of practice at the respective PHC center (**mean ± SD)	8.1 ± 7.2

### Reliability tests

Cronbach’s alpha value for the core, technological and learning readiness categories was 0.93. The alpha values for societal and policy readiness were 0.90 and 0.91, respectively.

### Readiness for e-Health

#### Core readiness

Regarding core readiness, the highest scoring items were those concerned with staff comfort using ICT for storing patient information, patient care, and education. On the other hand, the lowest scores were for staff involvement in planning. Details of the core readiness are presented in Table 2.

**Table 2:** Core e-Health readiness results for managers and HVWs at Khartoum state PHC centers (n = 262)

Item	Agree/strongly agree	Neutral	Disagree/strongly disagree	Mean
**Core Readiness**				
Organization has properly identified its needs.	52.1	20.5	**27.4**	2.20
Organization has properly prioritized its needs.	**48.8**	23.8	**27.3**	2.15
There is general dissatisfaction with current handling of issues that could be addressed through telehealth/eHealth.	58.8	19.1	**22.1**	2.43
Solutions other than telehealth/e-Health have been explored.	**36.7**	30.5	**32.8**	**1.92**
Awareness of ICT and internet’s role in healthcare exists among the planners.	61.6	18.2	**20.2**	2.43
Awareness of ICT and internet’s role in addressing the prioritized needs exists among the planners.	60.5	19.2	**20.3**	2.44
There is general comfort in using ICT/internet among users of the proposed telehealth/e-Health project.	75.1	10.7	14.2	2.84
There is general comfort among staff in using ICT/internet for storing patient information.	81.7	8.4	9.9	3.06
There is general comfort among staff in using ICT/internet for the purpose of patient care and education.	80.8	8.5	10.8	2.99
All the policymakers and senior administrators trust new technology as a solution to the identified problems.	64.8	21.5	13.8	2.67
All the staff members trust new technology as a solution to the identified problems.	62.2	22.9	14.9	2.63
There are plans in place to increase staff’s trust and confidence in the new technology.	**40.9**	24.3	**34.7**	**1.99**
An individual or a group has taken responsibility for planning for the new telehealth/e-Health project.	**31.4**	24.9	**43.7**	**1.73**
All the user groups among staff and other stakeholders have been involved in planning for the new telehealth/e-Health project.	**28.4**	21.8	**49.8**	**1.60**
There is an appropriate plan for implementation of telehealth/e-Health Initiative.	**38.2**	24.7	**37.1**	**1.90**
The telehealth/e-Health implementation plan includes proper budgeting and identification of resources.	**47.1**	21.2	**31.7**	2.11
There is an appropriate plan for evaluation of telehealth/e-Health initiatives including options for external evaluation.	**37.5**	26.4	**36.0**	**1.94**
The technology is appropriate according to the conditions within the center.	54.8	18.9	**26.3**	2.28
There is a willingness among staff to implement the technology for its intended purpose.	79.4	13.7	6.9	2.96
Integration of technology with the current services has been considered in the planning process.	**41.2**	29.8	**29.0**	2.04
There is a plan in place to integrate telehealth/ e-Health with the current services.	**36.4**	27.6	**36.0**	**1.89**

Flags: less than 50% agree/strongly agree; more than 30% neutral; more than 20% disagree/strongly disagree. Mean less than 2

#### Technological readiness

The majority of the participating PHC managers (51.7%) responded that the quality of connections is not appropriate for the proposed use in e-Health. Although “Required ICT (telephone/internet/bandwidth) is easily available for the institutions involved” scored the highest mean in the category, only 43.8% of the managers agreed, and more than 20% showed disagreement. Details of PHC managers’ technological readiness are summarized in Table 3.

**Table 3:** e-Health technological readiness results for managers at Khartoum state PHC centers (n = 26)

Item	Agree/strongly agree	Neutral	Disagree/strongly disagree	Mean
**Technological Readiness**				
Speed of connections is appropriate for the proposed use.	**34.5**	27.6	**37.9**	**1.93**
Quality of connections is appropriate for the proposed use	**31.0**	17.2	**51.7**	**1.66**
Service/support is available within a reasonable time frame for the proposed use.	**31.0**	27.6	**41.4**	**1.69**
Local support is proficient to address most of the problems related to the proposed use.	**34.5**	**34.5**	**31.0**	**1.97**
Hardware and software required for the proposed project are readily available.	**27.6**	**37.9**	**34.5**	**1.83**
Hardware and software required for the proposed project are readily affordable.	**41.4**	**31.0**	**27.6**	2.03
Required ICT (telephone/internet/bandwidth) is easily available for the institution.	**48.3**	27.6	**24.1**	2.10
Required ICT (telephone/internet/bandwidth) is easily available for the institutions involved.	**44.8**	**34.5**	**20.7**	2.17
Programs are in place to train the users for the proposed project.	**37.9**	27.6	**34.5**	2.03
Manpower is in place to train the users for the proposed project.	**34.5**	41.4	**24.1**	2.03

Flags: less than 50% agree/strongly agree; more than 30% neutral; more than 20% disagree/strongly disagree. Mean less than 2

#### Learning readiness

In terms of PHC workers’ learning readiness, the item “Personnel and programs are in place for ICT/Internet training” scored the lowest mean in the category (1.9). Although the statement “Programs exist for continuous education” had the highest mean, more than 30% of the HCPs disagreed with it. Details of the PHC workers’ learning readiness are illustrated in Table 4.

**Table 4:** e-Health learning readiness results for HCWs at Khartoum state PHC centers (n = 233)

Item	Agree/strongly agree	Neutral	Disagree/strongly disagree	Mean
**Learning Readiness**				
Personnel and programs are in place for ICT/Internet training.	**41.8**	15.9	**42.2**	**1.90**
Programs exist for continuous education.	**48.7**	19.8	**31.5**	2.16
ICT/Internet is readily used in continuous education	**46.6**	23.3	**30.2**	2.13
Programs are in place to use ICT/Internet for continuous education.	**42.2**	22.8	**34.9**	2.01
There is a plan in place to involve healthcare workers in the planning of new telehealth/e-Health interventions.	**44.0**	24.1	**31.9**	2.07
There is a plan in place to involve healthcare workers in the implementation of new tele-health/e-Health interventions.	**42.9**	**42.5**	**32.6**	2.08

Flags: less than 50% agree/strongly Agree; more than 30% neutral; more than 20% disagree/strongly sisagree. Mean less than 2

#### Societal readiness

In terms of societal readiness, it is worth noting that the item concerning gender-based benefits of e-Health received the highest mean score (2.85). On the other hand, items addressing ICT use for inter-institutional referrals and communication with the local communities received the lowest mean scores: 1.71 and 1.77, respectively. A summary of societal readiness results is provided in Table 5.

**Table 5:** e-Health societal readiness results for managers and HCWs at Khartoum state PHC centers (n = 262)

Item	Agree/strongly agree	Neutral	Disagree/strongly disagree	Mean
**Societal Readiness**				
Staff regularly use ICT/Internet to communicate with staff at the other health institutions of the region.	**45.2**	12.3	**42.5**	**1.89**
Staff regularly use ICT/Internet to communicate with local communities and clients.	**38.2**	15.8	**45.9**	**1.77**
Other institutions have planned to go through e-readiness assessment.	**30.4**	**35.4**	**34.2**	**1.87**
Material on locally relevant health issues is shared between this institution and other institutions.	50.6	24.3	**25.1**	2.24
The relevant material is available in language(s) easily understood by all the concerned staff and other users of information.	60.5	18.8	**20.7**	2.41
A referral system is available between this institution and other healthcare institutions to provide patient care in certain specialties.	65.6	13.1	**21.2**	2.50
ICT is currently used for referrals between this institution and other healthcare institutions.	**35.3**	18.6	**46.1**	**1.71**
Both staff genders have equal and unrestricted access to the technology.	65.3	17.4	17.4	2.56
Staff from all levels gets direct benefit from the use of technology.	63.8	16.9	19.2	2.57
Use of ICT will benefit men and women equally in the society.	77.1	10.1	12.8	2.85
People from all socioeconomic strata get direct benefit from the use of technology.	70.4	12.7	16.9	2.72

Flags: Less than 50% agree/strongly agree; more than 30% neutral; more than 20% disagree/strongly disagree. Mean less than 2

#### Policy readiness

In terms of policy readiness, the lowest means were for reimbursement policies at the institutional level (1.66), and governmental level (1.62). Items discussing policy makers’ and politicians’ awareness of the benefits of ICT in health care institutions received the highest scores in the category. Policy readiness findings are presented in Table 6.

**Table 6:** e-Health policy readiness for managers and HCWs at Khartoum state PHC centers (n = 262)

Item	Agree/Strongly agree	Neutral	Disagree/Strongly disagree	Mean
**Policy Readiness**				
Government policies are in place to promote and manage use of telehealth/e-Health in healthcare institutions.	**40.6**	27.2	**32.2**	2.01
Institutional policies are in place to promote and manage use of telehealth/e-Health in your institution.	**31.9**	25.0	**43.1**	**1.76**
Government policies are in place to allow care provision in other jurisdictions through telehealth.	**35.5**	29.0	**35.5**	**1.90**
Institutional policies are in place to allow care provision in other jurisdictions through telehealth.	**30.3**	24.9	**44.8**	**1.72**
Government policies are in place to deal with liability issues.	54.5	29.2	16.3	2.41
Institutional policies are in place to deal with liability issues.	58.3	25.5	16.2	2.47
Government policies are in place to ensure proper reimbursement to the healthcare workers in your institution.	**28.7**	25.7	**45.6**	**1.62**
Institutional policies are in place to ensure proper reimbursement to the healthcare workers in your institution.	**29.0**	27.8	**43.2**	**1.66**
Politicians are generally aware of the benefits of ICT use in healthcare.	64.0	24.4	11.6	2.62
Politicians generally support the use of ICT in healthcare.	**49.2**	25.0	**25.8**	2.19
Policy makers are aware of the benefits of ICT in healthcare institutions.	69.7	18.8	11.5	2.70
Policy makers support the use of ICT in healthcare institutions.	52.1	24.5	**23.4**	2.26

Flags: less than 50% agree/strongly agree; more than 30% neutral; more than 20% disagree/strongly disagree. Mean less than 2

### Readiness subscales and total scores

Regarding the overall e-Health readiness subscales analysis, the highest readiness percentage was for core readiness (57%), followed by societal readiness (56.6%). Technological readiness, which is only assessed for managers, received the lowest readiness percentage of 48.8%. The overall readiness percentages for managers and health care workers were 52.8% and 55.3%, respectively. Details of the total readiness scale and subscales results are illustrated in Table 7.

**Table 7:** Subscales and total e-Health readiness scores for managers and health care workers at Khartoum state PHC centers (n = 262)

Subscale [Total]			Mean	Median (IQR)	Readiness percentage
Core Readiness [84]			47.9	51.0 (39.0,60.0)	**57.0%**
Manager			47.1	45.0 (39.5,58.5)	56.4%
HCP			48.0	51.0 (39.0,60.0)	57.1%
Learning Readiness* [24]			12.3	12.0 (8.0,18.00)	**51.3%**
Technological Readiness** [40]			19.5	18.0 (15.5,26.0)	**48.8%**
Societal Readiness [44]			24.9	26.0 (20.0,32.0)	**56.6%**
Manager			23.1	23.0 (16.0,31.5)	52.5%
HCP			25.1	27.0 (20.5,32.0)	57.0%
Policy Readiness [48]			25.2	26.0 (20.0,32.0)	**52.5%**
Manager			24.3	24.0 (19.5,30.5)	50.6%
HCP			25.3	26.0 (19.0,32.0)	52.7%
Total Readiness					
Manager [216]			114.0 ± 28.5	114.0 (100.0,130.5)	**52.8%**
HCP [200]			110.6 ± 38.1	112.0 (89.0,138.0)	**55.3%**

[Total] = Total category score. *Subscale for HCP only. **Subscale for managers only. Readiness percentage = mean score/total score. Flags: subscale readiness percentage less than 60%

### Factors associated with e-Health readiness

In a group-based comparison, total and core readiness scores showed significant differences according to the health workers’ occupation within the healthcare system, with *p*-values of 0.038 and 0.029, respectively. Among doctors with different levels of expertise, a significant difference was detected only in learning readiness scores (*p* value: 0.039).

Moreover, a significant difference was detected in the readiness scores among the 36 i PHC centers in both the total scale and all subscales except technological readiness. The respective *p*-values were < 0.001 for core, policy, and total readiness scores. In addition, *p* values of 0.008 and 0.001 were detected for societal and learning readiness, respectively. Another significant finding was the difference in total and learning readiness scores between the different center types, with *p* values of 0.035 and 0.027 respectively.

Other tested factors, including gender, age, educational level, employment status, job title at the respective PHC center, total years of practice, total years of experience at the respective center, and the center’s distance from the center of Khartoum State, showed no significant effect on the total and subscale readiness scores. Elaborated test results are provided in supplementary Tables 1 and 2 from supplementary file 4.

Furthermore, in the standard multiple regression model, no significant associations were detected between total readiness scores and tested factors. The model summary is provided in supplementary Table 3 from supplementary file 4.

## Discussion

In healthcare, e-readiness is defined as “the degree to which users, healthcare institutions, and the healthcare system itself are prepared to participate and succeed with e-Health implementation” [15]. Since e-Health endorsement demands a transformational change in the targeted health system [15], a crucial step to be considered prior to its adoption is readiness assessment [3, 14]. To our knowledge, this is the first study in Sudan and the North African region to assess readiness for e-Health implementation in the PHC setting.

Our study reports e-Health readiness scores of less than 60% for both managers and HCWs, and calls attention to a number of associated factors. Although low, the core readiness category was the closest to our designated cutoff point of 60%. Seeing that the items of this category reflect the need for change and awareness about e-Health, it is perhaps the first category that health administrators should address as it was deemed the most influential of e-Health readiness parameters [18]. In our study, core readiness was associated with the health workers’ job title, which can be explained by the different educational curricula and training backgrounds for different groups. The favorable results of the Gezira Family Medicine Project support this. Through the deployment of multimodal e-Health training methods for family physicians, this project led to the successful implementation of a Telemedicine project at the PHC level in Gezira State, Sudan [19].

Developing countries are particularly vulnerable to barriers pertaining to technological readiness, including equipment, cost, and infrastructure [20, 21]. It follows that our findings of poor technological readiness were predictable and raise concerns about the situation in the other relatively under-served states. Sudan is reported to have moderate to high levels of inequalities in health resource and facility distribution among the 18 states, based on data from the 2016 Sudan Health Statistical Report [22]. This effect of regional inequalities in health resource distribution was also noted in a study conducted in Afghanistan, with managers in two different cities within the same country asymmetrically reflecting low and high levels of technological readiness [23]. Additionally, the significant difference between readiness scores of different PHC centers reported in this study reveals an additional layer of inequality within Khartoum state. On the other hand, over 70% of the respondents reported that women and men would benefit equally from the implementation of e-Health. This is an encouraging finding, as addressing gender-related inequities helps increase the benefit of e-Health projects by reducing the digital divide that is more pronounced in developing countries [14].

When studying the overall readiness scores in our study, the figures reflect a relatively higher readiness score when compared to a 30% total score reported from a study in Egyptian hospitals [23]. The soundness of this comparison is limited by the fact that we are comparing results from primary health care facilities to those from secondary and tertiary facilities, which reflects the paucity of relevant research in the region. Examining the relative readiness in the different categories is crucial, even when all categories have scores that fall below 60% of the total score. We conclude that core and societal readiness scores are relatively good. While a similar study in Afghanistan hospitals reported high scores of core readiness [23], another study in Mauritius [24] identified core and societal readiness as the lowest scoring categories. On the other hand, technological readiness received the lowest score in our study, while it received high scores in the studies from Afghanistan [23] and Mauritius [24].

### Policy and practice implications

Sudan started the PHC approach in 1976 as a part of a five-year developmental program [25]. The year 2005 saw the first initiative to integrate e-Health as a part of Sudan’s health system, and over the next years, several overlapping efforts called attention to the evident lack of coordination [26]. Over a decade later, Sudan’s national health policy has yet to include e-Health [27]. This study hopes to contribute to future policies by providing a much-needed assessment of the readiness of potential providers of e-Health.

In a recent systematic review, cost and reimbursement issues appeared to be the most frequently listed barriers to telemedicine programs adoption [23]. When looking at the Sudanese context, two financial issues present themselves: first, providing the required logistics using resources from an already underfunded and overburdened health system. Second, relying mostly on out-of-pocket expenditure to finance healthcare with a limited contribution from national insurance [18], meaning that a decision to utilize and pay for e-Health lies largely within the hands of patients. In our study, reimbursement policies received the lowest score in their category. Hence, HCWs must be reassured of proper and timely reimbursement for the smooth implementation of e-Health programs.

In addition to the issues highlighted above, this study calls attention to a number of specific factors to be addressed by policymakers. Limited workers’ involvement in e-Health planning, as reflected in our study, was reported in other studies as well [23]. Workers’ involvement is agreed to be crucial for preparing HCWs for e-Health and for better implementation of the planned programs [28, 29]. All items in the learning readiness category received a score below 50% of the maximum score. Therefore, we share the recommendations of Saleh et al. to invest in capacity building efforts geared toward e-Health training for HCWs [17], especially that less than 40% of the health workforce in our study were bachelor’s degree holders.

As of the time of writing this article, Khatoum, where this study took place, is laboring under a violent armed conflict [30]. E–health is now informally implemented in the wake of the ongoing war, allowing physicians to provide services beyond their physical reach. We predict a change in the readiness scores if a post-war assessment is carried out. While the core readiness scores might increase as a result of increasing awareness of the importance of e-Health, technological readiness might further deteriorate as many assets were looted or destroyed in the armed conflict. This emphasizes the need for a nation-wide assessment. The questionnaire used in this study is recommended for helping institutions identify weaknesses and set clear bases for planning e-Health projects beyond the dilemma of comparison [14].

### Limitations

Our study has several limitations. First, our study focused only on Khartoum state PHC centers. However, Khartoum is the capital state and has the highest concentration of PHC centers and HCWs. This puts Khartoum at a technological advantage and makes it the most eligible target for future e-Health endeavors. This still limits the generalizability of the findings at the national level but allows us to make predictions about the readiness status in the remaining less-advantaged states, where readiness scores might be even lower. Another limitation is the failure of the backward translation of the study tool due to the lack of experts and translation boards specialized in health-related matters. However, it is worth noting that backward translation was criticized for heavily relying on literal translations and implying that a perfect equivalence is attainable [31]. Another limitation is the subjectivity of the self-reported assessment tool to the participants’ perspectives, which necessitates more objective methods to support its findings. Moreover, the unavailability of study participants and their unwillingness to fill out the long study questionnaire, which takes 10 to 15 min to complete properly, constituted a limitation of the study. In anticipation of this problem, we employed a direct contact approach whenever possible to increase the response rate, an approach found to result in significantly higher response rates [32]. Despite our efforts, our response rate (79%) hindered our study from attaining the minimum calculated sample size.

## Conclusion

This study reports low levels of e-Health readiness as reported by managers and HCWs in Khartoum state PHC. Despite the agreement on the urgent need for e-Health implementation, project planners must be aware of the obstacles and threats that can accompany these programs if not planned, executed, and monitored carefully. Furthermore, in such projects, special attention must be given to addressing various types of health inequities and inequalities, to ensure that these projects will contribute to fostering accessibility to health services and narrowing the digital divide, rather than widening it.

### Electronic supplementary material

Below is the link to the electronic supplementary material.

Supplementary Material 1

Supplementary Material 2

Supplementary Material 3

Supplementary Material 4

Supplementary Material 5

## Data Availability

All data generated or analyzed during this study are included in this published article [and its supplementary information files, supplementary file 2].

## Abbreviations

e-Health: Electronic Health
PHC: Primary Health Care
HCW: Health Care Worker
WHO: World Health Organization
EMRs: Electronic medical records
SPSS: Statistical Package for Social Sciences
SD: Standard Deviation
