# Supplementary material for: Readiness of managers and health care workers for e-Health: a cross-sectional study in Khartoum primary health care centers, Sudan
Source: BMC Health Serv Res. 2023 Dec 12;23:1399. doi: 10.1186/s12913-023-10450-6 (PMC10717329; doi:10.1186/s12913-023-10450-6)
Supplement: Supplementary file 1 — Supplementary Material 1 [file 12913_2023_10450_MOESM1_ESM.docx]

**Supplementary file 1: Selected PHC centers and distance from Khartoum State center**


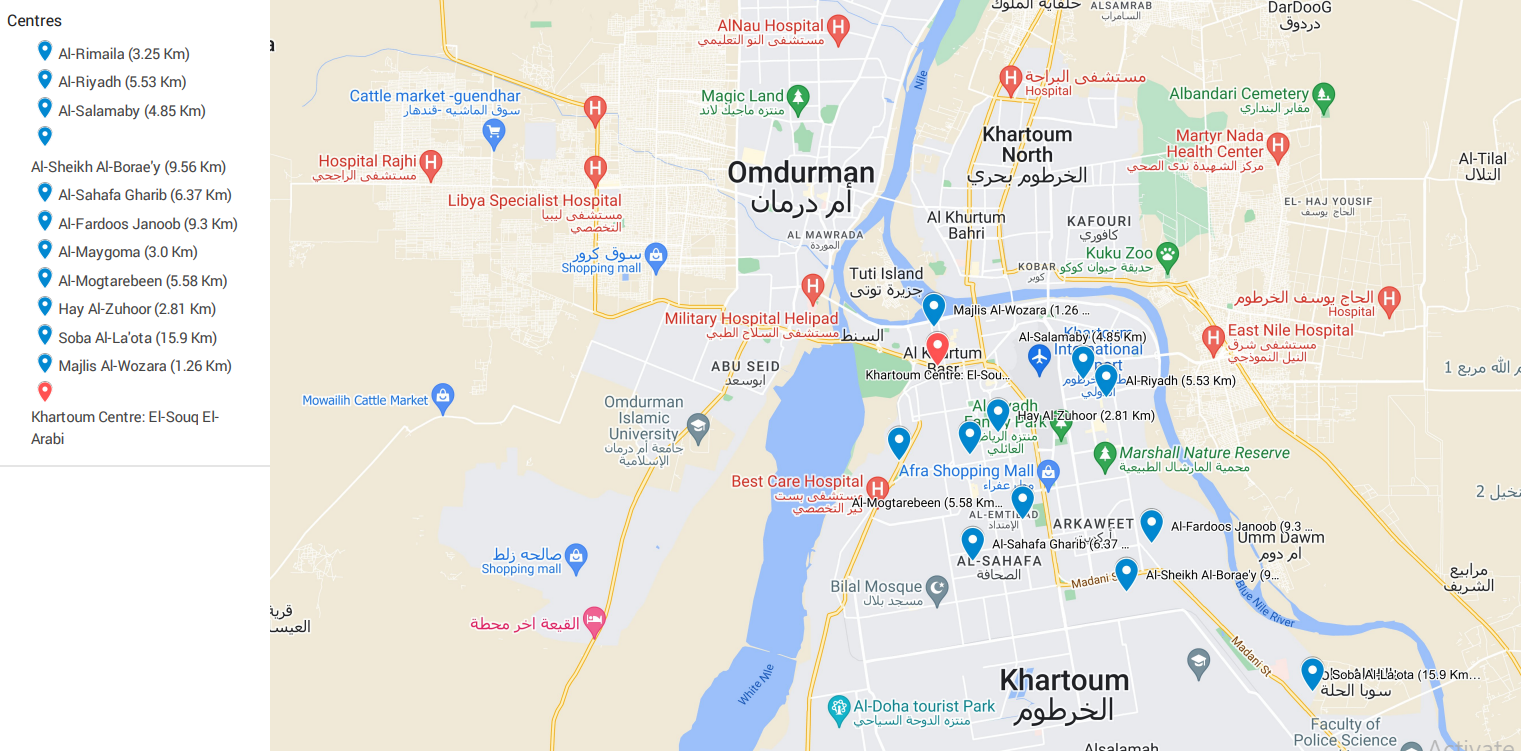


***Supplementary Figure 1*** Selected PHC centers from Khartoum locality and distance from Khartoum center


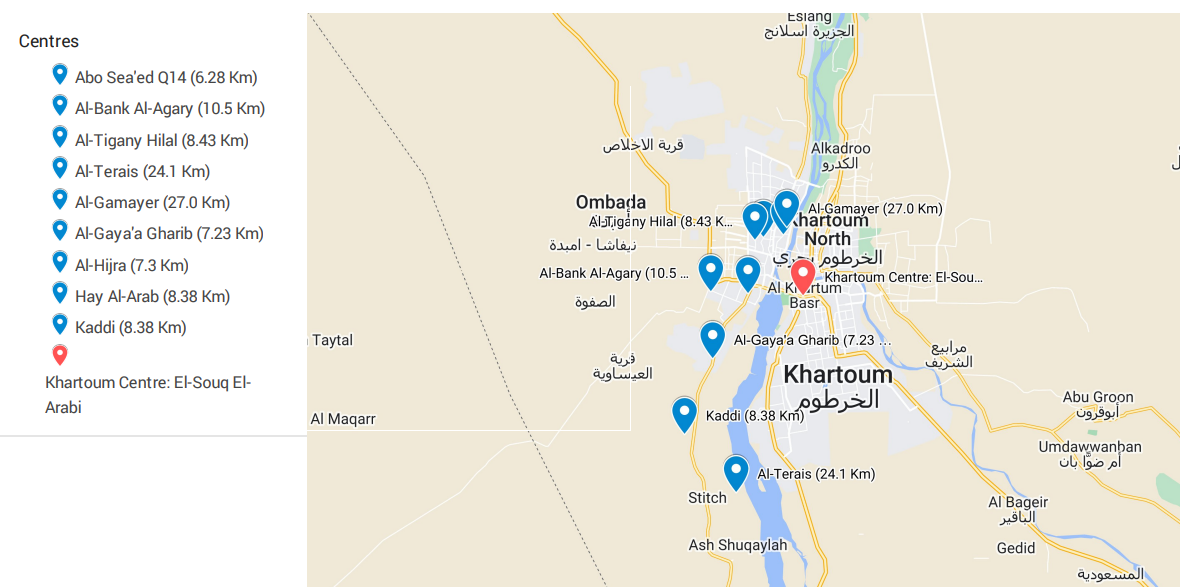


***Supplementary Figure 2*** Selected PHC centers from Omdurman locality and distance from Khartoum center


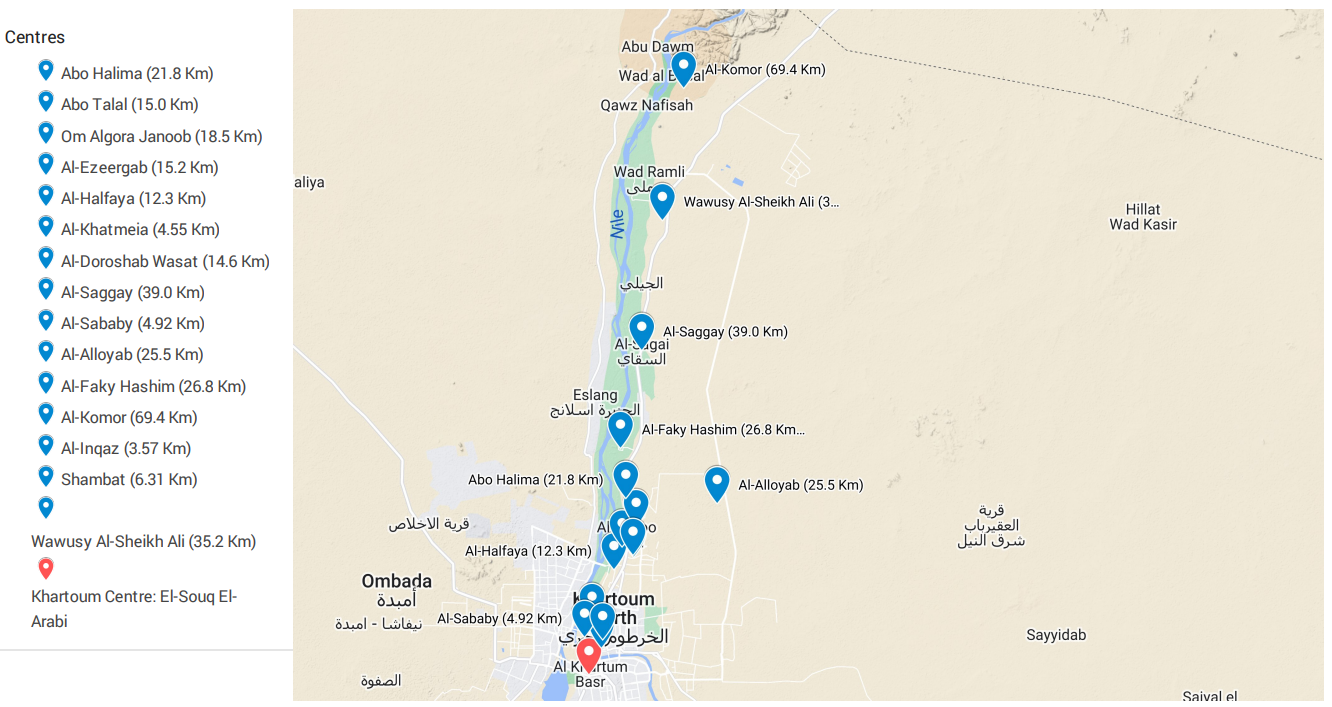


***Supplementary Figure 3*** Selected PHC centers from Bahri locality and distance from Khartoum center
