# Supplementary material for: Readiness of managers and health care workers for e-Health: a cross-sectional study in Khartoum primary health care centers, Sudan
Source: BMC Health Serv Res. 2023 Dec 12;23:1399. doi: 10.1186/s12913-023-10450-6 (PMC10717329; doi:10.1186/s12913-023-10450-6)
Supplement: Supplementary file 2 — Supplementary Material 2 [file 12913_2023_10450_MOESM2_ESM.docx]

***Supplementary file 2:*** *Data collection tool for the assessment of managers and health care workers readiness for e-health*

| **Num.** | **Statement** |  |  |  |  |  |
| --- | --- | --- | --- | --- | --- | --- |
|  | **Core-Readiness** |  |  |  |  |  |
|  | Organization has properly identified its needs. |  |  |  |  |  |
|  | Organization has properly prioritized its needs. |  |  |  |  |  |
|  | There is general dissatisfaction with current handling of issues that could be addressed through telehealth/e-health. |  |  |  |  |  |
|  | Solutions other than telehealth/e-health have been explored. |  |  |  |  |  |
|  | Awareness of ICT and internet’s role in healthcare exists among the planners. |  |  |  |  |  |
|  | Awareness of ICT and internet’s role in addressing the prioritized needs exists among the planners. |  |  |  |  |  |
|  | There is general comfort in using ICT/internet among users of the proposed telehealth/e-health project. |  |  |  |  |  |
|  | There is general comfort among staff in using ICT/internet for storing patient information. |  |  |  |  |  |
|  | There is general comfort among staff in using ICT/internet for the purpose of patient care and education. |  |  |  |  |  |
|  | All the policymakers and senior administrators trust new technology as a solution to the identified problems. |  |  |  |  |  |
|  | All the staff members trust new technology as a solution to the identified problems. |  |  |  |  |  |
|  | There are plans in place to increase staff’s trust and confidence in the new technology. |  |  |  |  |  |
|  | An individual or a group has taken responsibility for planning for the new telehealth/e-health project. |  |  |  |  |  |
|  | All the user groups among staff and other stakeholders have been involved in planning for the new telehealth/e-health project. |  |  |  |  |  |
|  | There is an appropriate plan for implementation of telehealth/e-health  Initiative. |  |  |  |  |  |
|  | The telehealth/e-health implementation plan includes proper budgeting and identification of resources. |  |  |  |  |  |
|  | There is an appropriate plan for evaluation of telehealth/e-health initiative including option for external evaluation. |  |  |  |  |  |
|  | The technology is appropriate according to the conditions within the center. |  |  |  |  |  |
|  | There is a willingness among staff to implement the technology for its intended purpose. |  |  |  |  |  |
|  | Integration of technology with the current services has been considered in the planning process. |  |  |  |  |  |
|  | There is a plan in place to integrate telehealth/e-health with the current services. |  |  |  |  |  |
|  | **Technological Readiness** |  |  |  |  |  |
|  | Speed of connections is appropriate for the proposed use. |  |  |  |  |  |
|  | Quality of connections is appropriate for the proposed use |  |  |  |  |  |
|  | Service/support is available within a reasonable time frame for the proposed use. |  |  |  |  |  |
|  | Local support is proficient to address most of the problems related to the proposed use. |  |  |  |  |  |
|  | Hardware and software required for the proposed project are readily available. |  |  |  |  |  |
|  | Hardware and software required for the proposed project are readily affordable. |  |  |  |  |  |
|  | Required ICT (telephone/internet/bandwidth) is easily available for the institution. |  |  |  |  |  |
|  | Required ICT (telephone/internet/bandwidth) is easily available for the institutions involved. |  |  |  |  |  |
|  | Programs are in place to train the users for proposed project. |  |  |  |  |  |
|  | Manpower is in place to train the users for proposed project. |  |  |  |  |  |
|  | **Learning Readiness** |  |  |  |  |  |
|  | Personnel and programs are in place for ICT/Internet training. |  |  |  |  |  |
|  | Programs exist for continuous education. |  |  |  |  |  |
|  | ICT/Internet is readily used in continuous education |  |  |  |  |  |
|  | Programs are in place to use ICT/Internet for continuous education. |  |  |  |  |  |
|  | There is a plan in place to involve healthcare providers in the planning of new telehealth/e-health interventions. |  |  |  |  |  |
|  | There is a plan in place to involve healthcare providers in the implementation of new telehealth/e-health interventions. |  |  |  |  |  |
|  | **Societal Readiness** |  |  |  |  |  |
|  | Staff regularly uses ICT/Internet to communicate with staff at the other health institutions of the region. |  |  |  |  |  |
|  | Staff regularly uses ICT/Internet to communicate with local community and clients. |  |  |  |  |  |
|  | Other institutions have planned to go through e-readiness assessment. |  |  |  |  |  |
|  | Material on locally relevant health issues is shared between this institution and other institutions. |  |  |  |  |  |
|  | The relevant material is available in language(s) easily understood by all the concerned staff and other users of information. |  |  |  |  |  |
|  | A referral system is available between this institution and other healthcare institutions to provide patient care in certain specialities. |  |  |  |  |  |
|  | ICT is currently used for referrals between this institution and other healthcare institutions. |  |  |  |  |  |
|  | Both staff genders have equal and unrestricted access to the technology. |  |  |  |  |  |
|  | Staff from all levels get direct benefit from the use of technology. |  |  |  |  |  |
|  | Use of ICT will benefit men and women equally in the society. |  |  |  |  |  |
|  | People from all socioeconomic strata get direct benefit from the use of technology. |  |  |  |  |  |
|  | **Policy Readiness** |  |  |  |  |  |
|  | Government policies are in place to promote and manage use of telehealth/e-health in healthcare institutions. |  |  |  |  |  |
|  | Institutional policies are in place to promote and manage use of telehealth/e-health in your institution. |  |  |  |  |  |
|  | Government policies are in place to allow care provision in other jurisdictions through telehealth. |  |  |  |  |  |
|  | Institutional policies are in place to allow care provision in other jurisdictions through telehealth. |  |  |  |  |  |
|  | Government policies are in place to deal with liability issues. |  |  |  |  |  |
|  | Institutional policies are in place to deal with liability issues. |  |  |  |  |  |
|  | Government policies are in place to ensure proper reimbursement to the healthcare providers in your institution. |  |  |  |  |  |
|  | Institutional policies are in place to ensure proper reimbursement to the healthcare providers in your institution. |  |  |  |  |  |
|  | Politicians are generally aware of the benefits of ICT use in healthcare. |  |  |  |  |  |
|  | Politicians generally support the use of ICT use in healthcare. |  |  |  |  |  |
|  | Policy makers are aware of the benefits of ICT in healthcare institutions. |  |  |  |  |  |
|  | Policy makers support the use of ICT in healthcare institutions. |  |  |  |  |  |
