# Supplementary material for: Readiness of managers and health care workers for e-Health: a cross-sectional study in Khartoum primary health care centers, Sudan
Source: BMC Health Serv Res. 2023 Dec 12;23:1399. doi: 10.1186/s12913-023-10450-6 (PMC10717329; doi:10.1186/s12913-023-10450-6)
Supplement: Supplementary file 4 — Supplementary Material 4 [file 12913_2023_10450_MOESM4_ESM.docx]

**Supplementary File 4: Factors associated with eHealth readiness**

***Supplementary Table 1*** Factors associated with eHealth readiness (Bivariate analysis: categorical variables sum ranks)

| **Item** | **Core Readiness** | **Societal Readiness** | **Policy Readiness** | **Technological Readiness** | **Learning Readiness** | **Total Readiness score** |
| --- | --- | --- | --- | --- | --- | --- |
| **Gender** | | | | | | |
| Male | 142.41 | 146.58 | 141.49 | 16.64 | 118.42 | 143.51 |
| Female | 128.93 | 128.18 | 128.56 | 14.48 | 116.18 | 128.74 |
| P value | 0.299 | 0.156 | 0.322 | 0.557 | 0.859 | N/A |
| **Employment status at the respective PHC center** | | | | | | |
| full time | 125.01 | 126.19 | 125.53 | 15.83 | 110.93 | 125.27 |
| part time | 136.66 | 134.40 | 134.18 | 13.64 | 121.50 | 136.16 |
| P value | 0.233 | 0.400 | 0.374 | 0.499 | 0.252 | 0.265 |
| **HCP vs Managers** | | | | | | |
| HCP | 132.73 | 133.72 | 132.38 | 0.00 | 117.00 | 131.13 |
| manager | 121.64 | 113.69 | 120.00 | 15.00 | 0.00 | 134.45 |
| P value | 0.457 | 0.179 | 0.405 | N/A | N/A | 0.824 |
| **Education** | | | | | | |
| Diploma | 51.15 | 59.01 | 48.53 | 4.00 | 46.00 | 50.72 |
| Bachelor’s degree | 49.67 | 50.12 | 62.26 | 5.00 | 50.89 | 53.69 |
| Others | 64.40 | 55.58 | 61.10 | 5.20 | 58.45 | 62.90 |
| P value | 0.090 | 0.571 | 0.110 | 0.923 | 0.157 | N/A |
| **Occupation** | | | | | | |
| Doctor | 109.79 | 102.65 | 109.86 | 13.19 | 94.79 | 112.90 |
| Pharmacist | 116.18 | 109.59 | 103.43 | N/A | 100.39 | 105.11 |
| Nurse | 135.80 | 128.03 | 133.30 | 10.50 | 114.37 | 132.05 |
| Lab technician | 103.58 | 108.32 | 112.17 | N/A | 92.95 | 102.82 |
| Administration/Finance | 136.63 | 140.07 | 129.82 | 13.67 | 126.79 | 137.79 |
| Midwife | 162.43 | 154.61 | 159.11 | 15.00 | 138.85 | 164.11 |
| Vaccination technician | 117.79 | 139.00 | 122.71 | N/A | 93.21 | 119.14 |
| Nutritionist | 76.75 | 115.08 | 142.50 | N/A | 103.42 | 99.25 |
| P value | **0.029** | 0.050 | 0.205 | 0.928 | 0.062 | **0.038** |
| **Doctors’ classification** | | | | | | |
| General Practitioner | 22.18 | 20.82 | 21.79 | 11.63 | 11.04 | 20.65 |
| Registrar | 22.61 | 23.82 | 25.58 | 9.75 | 16.50 | 25.44 |
| Specialist | 26.63 | 27.69 | 22.25 | 3.75 | 21.00 | 24.13 |
| P value | 0.683 | 0.227 | 0.467 | 0.222 | **0.039** | 0.429 |
| **PHC center** | | | | | | |
| Abo Halima | 90.88 | 122.25 | 127.83 | 28.50 | 72.67 | 111.50 |
| Abo Sea'ed Q14 | 157.95 | 138.00 | 158.25 | 2.00 | 143.22 | 153.45 |
| Abo Talal | 127.25 | 131.33 | 150.33 | 23.50 | 116.70 | 146.50 |
| Al-Ezeergab | 140.80 | 151.60 | 188.80 | 8.50 | 134.44 | 158.45 |
| Al-Bank Al-Agary | 151.00 | 129.56 | 163.22 | 21.00 | 124.75 | 154.00 |
| Al-Tigany Hilal | 73.71 | 52.21 | 81.86 | 19.50 | 46.75 | 60.29 |
| Al-Terais | 89.50 | 124.81 | 135.19 | 27.00 | 86.14 | 108.81 |
| Al-Halfaya | 162.39 | 137.17 | 158.39 | 13.50 | 111.75 | 152.94 |
| Al-Khatmeia | 142.39 | 125.39 | 125.11 | 19.50 | 125.13 | 140.22 |
| Al-Doroshab Wasat | 170.58 | 149.25 | 156.58 | 18.00 | 159.40 | 164.42 |
| Al-Rimaila | 143.13 | 141.63 | 142.75 | 23.50 | 129.07 | 147.88 |
| Al-Riyadh | 91.05 | 67.80 | 56.25 | 10.50 | 63.89 | 62.35 |
| Al-Saggay | 144.39 | 150.33 | 138.28 | 13.50 | 133.13 | 150.72 |
| Al-Sheikh Al-Borae'y | 88.43 | 166.64 | 51.07 | 4.50 | 78.25 | 89.64 |
| Al-Sababy | 24.93 | 22.07 | 35.79 | 1.00 | 31.83 | 19.57 |
| Al-Sahafa Gharib | 63.44 | 131.11 | 99.72 | 23.50 | 65.31 | 80.44 |
| Al-Alloyab | 135.25 | 112.13 | 57.88 | 23.50 | 99.67 | 105.38 |
| Al-Faky Hashim | 150.39 | 103.56 | 99.81 | 3.00 | 134.75 | 114.78 |
| Al-Gamayer | 136.50 | 131.44 | 113.56 | 4.50 | 114.25 | 126.44 |
| Al-Gaya'a Gharib | 75.78 | 80.89 | 89.83 | 13.50 | 80.63 | 74.94 |
| Al-Komor | 140.60 | 139.60 | 120.90 | 6.00 | 132.50 | 135.80 |
| Al-Maygoma | 110.94 | 124.28 | 131.28 | 10.50 | 120.69 | 121.72 |
| Al-Mogtarebeen | 190.92 | 131.50 | 183.00 | 11.50 | 156.75 | 183.08 |
| Al-Inqaz | 106.40 | 120.00 | 168.80 | 26.00 | 115.13 | 132.40 |
| Hay Al-Zuhoor | 137.75 | 139.38 | 92.13 | 8.50 | 101.79 | 120.25 |
| Soba Al-La'ota | 113.39 | 139.00 | 108.06 | 13.50 | 95.44 | 116.28 |
| Kaddi | 240.10 | 230.70 | 214.40 | 28.50 | 187.00 | 241.80 |
| Wawusy Al-Sheikh Ali | 183.38 | 171.25 | 178.75 | 17.00 | 170.86 | 186.13 |
| Om Algora Janoob | 169.83 | 176.44 | 167.00 | N/A | 156.56 | 179.44 |
| Al-Salamaby | 159.63 | 151.38 | 150.56 | N/A | 138.88 | 156.94 |
| Al-Fardoos Janoob | 155.19 | 169.63 | 169.94 | N/A | 143.75 | 164.88 |
| Al-Hijra | 118.00 | 132.25 | 147.08 | N/A | 96.42 | 121.33 |
| Hay Al-Arab | 32.75 | 93.75 | 38.25 | N/A | 31.75 | 35.75 |
| Shambat | 160.14 | 151.57 | 151.64 | N/A | 152.93 | 163.07 |
| Majlis Al-Wozara | 168.25 | 155.25 | 169.06 | N/A | 167.38 | 176.88 |
| P value | **0.000** | **0.008** | **0.000** | 0.440 | **0.001** | **0.000** |
| **Center type** | | | | | | |
| 10 Packages | 126.12 | 128.81 | 129.42 | 13.72 | 112.43 | 127.69 |
| Reference (More than 10 Packages) | 132.17 | 129.33 | 127.43 | 17.9 | 115.13 | 129.16 |
| Private | 168.50 | 168.94 | 170.09 | N/A | 160.41 | 178.94 |
| P value | 0.108 | 0.124 | 0.099 | 0.300 | **0.027** | **0.035** |
| **Locality** |  |  |  |  |  |  |
| Khartoum | 126.41 | 136.01 | 120.72 | 13.06 | 112.83 | 126.11 |
| Omdurman | 123.92 | 122.39 | 131.81 | 16.57 | 105.50 | 123.60 |
| Bahri | 140.39 | 133.24 | 139.23 | 15.50 | 127.69 | 140.83 |
| P value | 0.282 | 0.518 | 0.230 | 0.685 | 0.112 | 0.248 |

Results for Mann-Whitney U and Kruskall-Wallis tests for binomial and polynomial group comparison, respectively. N/A: Not Available.

***Supplementary Table 2*** Factors associated with eHealth readiness (Bivariate analysis: numerical variables correlation)

| **Item** | Core Readiness | Societal Readiness | Policy Readiness | Technological Readiness | Learning Readiness | Total Readiness score |
| --- | --- | --- | --- | --- | --- | --- |
| **Age** | | | | | | |
| Correlation coefficient | -0.064 | 0.014 | -0.052 | -0.130 | -0.046 | -0.070 |
| P value | 0.333 | 0.835 | 0.435 | 0.537 | 0.511 | 0.294 |
| **Distance from Khartoum center** | | | | | | |
| Correlation coefficient | 0.100 | 0.116 | 0.071 | 0.056 | 0.071 | 0.098 |
| P value | 0.108 | 0.061 | 0.250 | 0.773 | 0.277 | 0.114 |
| **Number of health packages activated in the center** | | | | | | |
| Correlation coefficient | 0.53 | 0.010 | 0.111 | -0.115 | 0.117 | 0.080 |
| P value | 0.558 | 0.915 | 0.219 | 0.639 | 0.233 | 0.377 |
| **Overall years of expertise** | | | | | | |
| Correlation coefficient | -0.097 | -0.059 | -0.041 | -0.257 | -0.088 | -0.103 |
| P value | 0.124 | 0.348 | 0.512 | 0.196 | 0.184 | 0.100 |
| **Years of expertise in the Center** | | | | | | |
| Correlation coefficient | -0.005 | 0.004 | 0.015 | -0.031 | 0.000 | -0.012 |
| P value | 0.935 | 0.952 | 0.817 | 0.878 | 0.994 | 0.852 |

Results for Spearman's Rho correlation test.

***Supplementary Table 3*** Factors associated with eHealth readiness (Multivariate analysis: Standard multiple regression)

| **Variable** | **Standardized Beta coefficients** | **P-value** | **95% Confidence Interval** | | **Collinearity Statistics** | |
| --- | --- | --- | --- | --- | --- | --- |
|  |  |  | **Lower bound** | **Upper bound** | **Tolerance** | **VIF** |
| Gender | -.085 | .431 | -30.866 | 13.289 | .830 | 1.205 |
| Age | -.073 | .663 | -1.603 | 1.024 | .348 | 2.873 |
| Education | -.059 | .618 | -5.928 | 3.542 | .697 | 1.436 |
| Occupation | .126 | .233 | -1.305 | 5.305 | .885 | 1.130 |
| PHC center | .011 | .925 | -.794 | .874 | .765 | 1.307 |
| Locality | -.026 | .830 | -11.424 | 9.188 | .661 | 1.513 |
| Distance from Khartoum Center | .167 | .203 | -.273 | 1.266 | .572 | 1.749 |
| Center type | .164 | .158 | -3.971 | 24.138 | .728 | 1.373 |
| Number of health packages activated in the center | -.025 | .828 | -4.097 | 3.285 | .721 | 1.388 |
| Employment status at the respective PHC center | -.080 | .437 | -22.196 | 9.666 | .919 | 1.088 |
| Overall years of expertise | .014 | .933 | -1.243 | 1.353 | .345 | 2.902 |
| Years of expertise in the Center | -.068 | .585 | -1.618 | .918 | .631 | 1.585 |

Results for standard multiple regression analysis. VIF = Variance inflation factor.
